# ALPK2 Promotes Cardiogenesis in Zebrafish and Human Pluripotent Stem Cells

**DOI:** 10.1016/j.isci.2018.03.010

**Published:** 2018-04-09

**Authors:** Peter Hofsteen, Aaron Mark Robitaille, Nicholas Strash, Nathan Palpant, Randall T. Moon, Lil Pabon, Charles E. Murry

**Affiliations:** 1 Department of Pathology, School of Medicine, University of Washington, 850 Republican Street, Brotman Building Room 453, Seattle, WA 98109, USA; 2 Department of Bioengineering, School of Medicine, University of Washington, Seattle, WA 98109, USA; 3 Department of Medicine (Division of Cardiology), School of Medicine, University of Washington, Seattle, WA 98109, USA; 4 Center for Cardiovascular Biology, School of Medicine, University of Washington, Seattle, WA 98109, USA; 5 Department of Pharmacology, School of Medicine, University of Washington, Seattle, WA 98109, USA; 6 Institute for Stem Cell and Regenerative Medicine, School of Medicine, University of Washington, Seattle, WA 98109, USA; 7 Howard Hughes Medical Institute, University of Washington, Seattle, WA 98109, USA

**Keywords:** Piscine Cardiology, Stem Cell Research, Developmental Biology

## Abstract

Cardiac development requires coordinated biphasic regulation of the WNT/β-catenin signaling pathway. By intersecting gene expression and loss-of-function siRNA screens we identified Alpha Protein Kinase 2 (ALPK2) as a candidate negative regulator of WNT/β-catenin signaling in cardiogenesis. In differentiating human embryonic stem cells (hESCs), ALPK2 is highly induced as hESCs transition from mesoderm to cardiac progenitors. Using antisense knockdown and CRISPR/Cas9 mutagenesis in hESCs and zebrafish, we demonstrate that ALPK2 promotes cardiac function and cardiomyocyte differentiation. Quantitative phosphoproteomics, protein expression profiling, and β-catenin reporter assays demonstrate that loss of ALPK2 led to stabilization of β-catenin and increased WNT signaling. Furthermore, cardiac defects attributed to ALPK2 depletion can be rescued in a dose-dependent manner by direct inhibition of WNT signaling through the small molecule XAV939. Together, these results demonstrate that ALPK2 regulates β-catenin-dependent signaling during developmental commitment of cardiomyocytes.

## Highlights

• ALPK2 is expressed and regulated during hESC cardiomyocyte lineage determination
• Cardiac development in zebrafish embryos and hESCs requires ALPK2
• ALPK2 negatively regulates WNT signaling to promote cardiomyocyte differentiation

## Graphical Abstract

**Figure:**
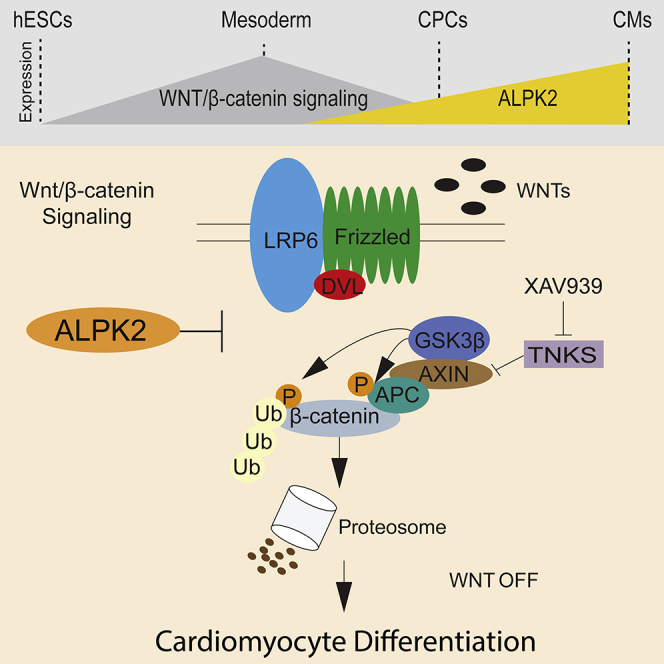

## Introduction

Directed differentiation from human embryonic stem cells (hESCs) toward cardiomyocytes serves as an *in vitro* model to elucidate regulatory mechanisms during human heart development (Hofsteen et al., 2016, Palpant et al., 2015a). Differentiation of cardiomyocytes requires temporal regulation of the WNT/β-catenin signal transduction pathway (Hofsteen et al., 2016, Lian et al., 2012, Naito et al., 2006, Palpant et al., 2015b, Ueno et al., 2007). Activation of WNT/β-catenin signaling is essential for the exit from pluripotency and mesoderm formation, whereas repression of the pathway is required for the transition toward the cardiomyocyte lineage (Davidson et al., 2012, Hofsteen et al., 2016, Palpant et al., 2015b). Studies have shown that modulation of the Wnt pathway is sufficient to direct cells through stage-specific transition during differentiation (Burridge et al., 2014, Lian et al., 2012). Thus, identifying regulators that inhibit WNT/β-catenin signaling is critical toward understanding human heart development.

WNT/β-catenin signaling is regulated by post-translational modifications of β-catenin (Gao et al., 2014, Moon et al., 2004). A “destruction complex” that contains scaffolding proteins and protein kinases phosphorylates β-catenin to display a motif that is recognized for ubiquitylation and degradation by the proteasome (Stamos and Weis, 2013). Lack of β-catenin phosphorylation activates WNT signaling (Stamos and Weis, 2013). Stabilized β-catenin shuttles into the nucleus and binds to transcription factors, notably TCF/LEF family members, to activate transcription of WNT target genes (Hsu et al., 1998, MacDonald et al., 2009). Continued activation of WNT/β-catenin signaling in the mesoderm represses cardiomyocyte fate and promotes endothelial and hematopoietic fate (Palpant et al., 2015b; wam\Woll et al., 2008). Thus, identifying regulators that inhibit WNT/β-catenin signaling is critical to control cell fate decisions during human heart development.

In the current study, by using combinatorial screening we identified a member of an atypical alpha protein kinase family member, alpha protein kinase 2 (ALPK2), as a cardiac developmental regulator and WNT/β-catenin signaling inhibitor. This protein family shares a highly conserved alpha protein kinase domain and, unlike conventional protein kinases, they are evolutionarily restricted to vertebrates (Middelbeek et al., 2010). There are six alpha kinases: eukaryotic elongation factor 2 kinase (eEF2K), TRP ion channel proteins (TRPM6 and TRPM7) as well as lymphocyte alpha kinase (LAK, or ALPK1), heart alpha kinase (HAK, or ALPK2), and muscle alpha kinase (MAK, or ALPK3), which were named from the tissues they were derived from (Drennan and Ryazanov, 2004, Middelbeek et al., 2010). ALPK2 has known roles in cancer by regulating cell cycle and DNA repair genes (Yoshida et al., 2012) and as a candidate regulator of hypertension (Chauvet et al., 2011), whereas its role during heart development has not been characterized. Our data indicate that one function of ALPK2 is to negatively regulate WNT/β-catenin signaling during cardiac development in hESCs and zebrafish.

## Results

### ALPK2 Is Expressed and Regulated during Cardiomyocyte Development

**Figure 1:**
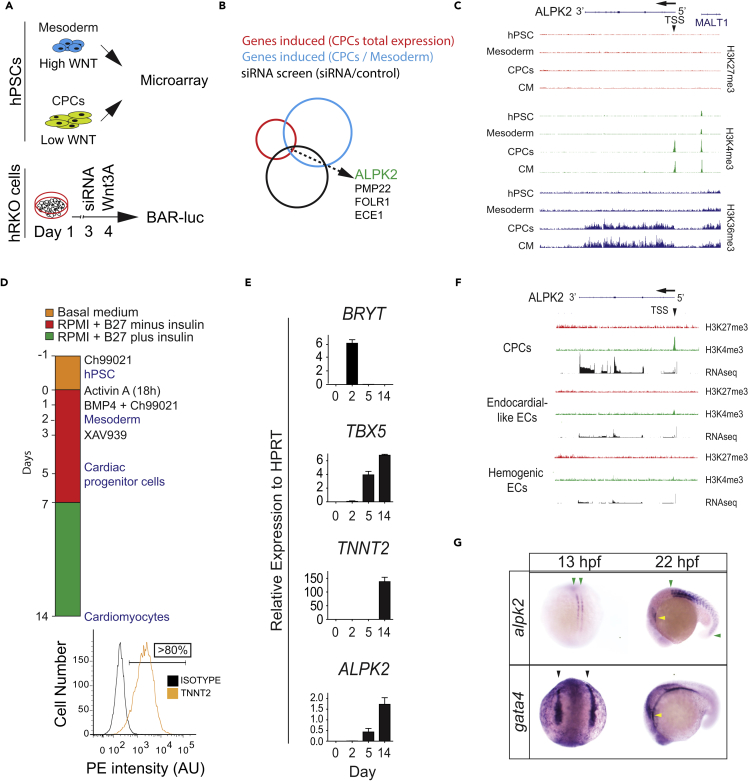
ALPK2 Identification and Expression Analyses (A) Schematic of combinatorial screening of RNA expression from hESC cardiomyocyte differentiation from human embryonic stem cells (hESCs) coupled with an siRNA screen using β-catenin-activated reporter (BAR)-transduced human RKO colon carcinoma cells (hRKO) stimulated with recombinant Wnt3A. (B) Venn diagram identifying Alpha Protein Kinase 2 (ALPK2). (C) Chromatin precipitation followed by deep sequencing (ChIP-Seq) for histone marks H3K4me3, H3K36me3, and H27K4me3 in hESC-derived cultures: hESC (day 0), mesoderm (day 2), cardiac progenitor cells (CPCs, day 5), and cardiomyocytes (day 14) (N = 2). (D) Protocol for high-density monolayer-directed differentiation of hESC-derived cardiomyocytes yielding a high percentage of cardiac troponin T (TNNT2)-positive cells by flow cytometry. (E) Quantitative RT-PCR analysis of markers of mesoderm (Brachyury T, *BRYT*), cardiac progenitor cells (T-box 5, *TBX5*), cardiomyocytes (*TNNT2)*, and *ALPK2* at days 0, 2, 5, 14 during cardiomyocyte differentiation. (F) RNA sequencing and ChIP-seq for H3K4me3 and H27K4me3 at the ALPK2 locus in CPCs, endocardial-like endothelial cells (EC), and hemogenic ECs (N = 2). (G) *In situ* hybridization of zebrafish *alpk2* and *gata4* at 13 hpf and 22 hr post fertilization (hpf, N = 22–36). Green arrowheads denote adaxial cells and primitive somites, black arrowheads mark the bilateral heart fields, and yellow arrowheads denote the primitive heart. Sample size N = 3–5 biological replicates, and data are displayed as mean ± SEM unless otherwise noted. See also Figure S1.

To identify negative regulators of the WNT/β-catenin signaling pathway, we conducted a combinatorial screen comparing previously published RNA expression from hESC-derived mesoderm and cardiac progenitor cells (CPCs) (Paige et al., 2012) with a small interfering RNA (siRNA) screen using human colorectal cancer cells (hRKO) carrying a β-catenin-activated reporter (BAR) driving luciferase (James et al., 2009) (Figure 1A). By intersecting these datasets we identified genes that were highly induced in CPCs that significantly increased BAR activity following siRNA knockdown. This analysis identified a previously unidentified putative cardiac and WNT/β-catenin signaling protein kinase, alpha protein kinase 2 (ALPK2; Figure 1B). ALPK2 was induced 68-fold in CPCs and increased BAR activity by 4-fold following siRNA-mediated knockdown.

We further explored the role of ALPK2 by surveying the epigenetic landscape over a staged time course of cardiomyocyte differentiation from hESCs (Paige et al., 2012). Epigenetic histone modification of chromatin marks H3K4me3 (active promoters) and H3K36me3 (RNA polymerase II activity) show that ALPK2 is epigenetically regulated as cells transition from mesoderm (day 2) to CPCs (day 5) and definitive cardiomyocytes (CMs, day 14) (Figure 1C). Of note, the repressive histone mark, H3K27me3, was not altered throughout the differentiation process (Figure 1C). We aimed to further characterize the expression of ALPK2 and assess its role during heart development. Toward this goal, hESCs were differentiated into cardiomyocytes using a high-density monolayer differentiation protocol (Palpant et al., 2015a). Differentiation of hESCs was induced with Activin A and bone morphogenetic protein 4 (BMP4) coupled with serial small molecule activation and inhibition of canonical WNT/β-catenin signaling (Figure 1D). This directed differentiation protocol typically yields high-purity cardiomyocytes (>80% cardiac troponin T; TNNT2^+^) as assessed by flow cytometry (Figure 1D). We analyzed transcript abundance in undifferentiated hESCs (day 0), mesodermal cells (day 2), CPCs (day 5), and cardiomyocytes (day 14). Expression of the pan-mesodermal marker Brachyury T (*BRYT*) peaked on day 2 and decreased to baseline levels by day 5. Following the peak of *BRYT* expression, the early cardiac marker T-box 5 (*TBX5*) could be detected at days 5 and 14. Lastly, the sarcomeric gene cardiac troponin T (*TNNT2*) was robustly expressed on day 14. These data indicated that normal mesoderm formation, CPC specification, and cardiomyocyte differentiation occurred. Analysis of *ALPK2* during this time course showed expression starting at day 5 with increasing levels at day 14 (Figure 1E, 2896-fold induction from pluripotency), demonstrating that ALPK2 is temporally expressed during specification of CPCs and cardiac commitment.

We subsequently compared the expression and epigenetic regulation of ALPK2 in three distinct mesodermal progenitor cell lineages (CPCs, endocardial-like endothelial cells [ECs], and hemogenic ECs) to decipher whether ALPK2 was expressed and regulated in non-cardiomyocyte lineages. These populations are generated by specifying distinct mesodermal sub-populations at the onset of differentiation by stimulation with different doses of Activin A and BMP4 as described in the Transparent Methods (Palpant et al., 2015b). We compared RNA sequencing and H3K4me3 and H3K27me3 histone modification at the ALPK2 locus and observed that ALPK2 was highly expressed and regulated in CPCs but not in endocardial-like ECs or hemogenic ECs (Figure 1F) (Palpant et al., 2017). In total, these data indicate ALPK2 is specifically regulated as hESCs differentiate toward the cardiomyocyte lineage.

Zebrafish (*Danio rerio*) are used as an *in vivo* model of heart development owing to the highly conserved developmental mechanisms and genome similarities to humans (Bakkers, 2011, Howe et al., 2013, Stainier and Fishman, 1994). In particular, sequence analyses show that among vertebrates, ALPK2 exhibits a high degree of conservation particularly within the alpha kinase domain (Figure S1). To visualize spatiotemporal expression during whole embryo development we analyzed zebrafish *alpk2* by *in situ* hybridization and compared its expression profile to a cardiac transcription factor, *gata4*. At 13 hr post fertilization (hpf), *gata4* is expressed in the bilateral heart, forming fields of pre-cardiac mesoderm, whereas *alpk2* is expressed in the precursor cells of slow twitch muscle fibers (adaxial cells) of the paraxial mesoderm. At 22 hpf, *gata4* and *alpk2* are co-expressed in the developing heart (Figure 1G). Thus ALPK2 is expressed during cardiac development *in vivo* and *in vitro*.

### Depletion of Alpk2 Impairs Zebrafish Heart Development

**Figure 2:**
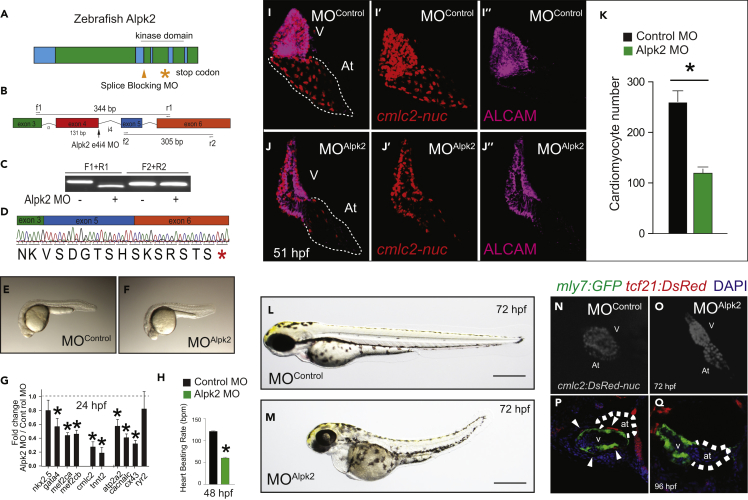
Alpk2 Is Essential for Zebrafish Cardiac Development (A) Zebrafish Alpk2 locus and targeted region by splice-blocking morpholino oligonucleotides (MO). * denotes stop codon. (B–D) RT-PCR and Sanger sequencing demonstrating splice-blocking MO caused alternative splicing resulting in a 131-base pair deletion and premature stop codon. * denotes stop codon. (E and F) Zebrafish injected with negative control MO (E) or Alpk2 MO (F) at 24 hr post fertilization (hpf). (G and H) Transcript analysis (G) and heart beating rate (H) of control and Alpk2 morphants at 24 and 48 hpf, respectively (N = 5, 15–22 pooled embryos per N). (I–O) Representative images of control and Alpk2 MO-injected hearts carrying a transgene for *cmlc2:DsRed-nuc* to quantify cardiomyocyte nuclei (K) at 51 hpf (N = 7–9). Red denotes *cmlc2:DsRed-nuc* and magenta is antibody staining for activated leukocyte cell adhesion molecule (ALCAM). Representative bright-field images of control MO (L) and Alpk2 MO (M) injected zebrafish at approximately 72 hpf. Representative images of control MO (N) and Alpk2 MO (O) injected zebrafish hearts carrying a transgene (*cmlc2:DsRed-nuc*) to denote cardiac morphology at approximately 72 hpf. (P and Q) Confocal images of control and *Alpk2* morphant fish carrying *myl7:GFP* and *tcf21:DsRed* at 96 hpf. White arrowheads denote *tcf21+* epicardial cells on the heart ventricle. N = 3; independent biological replicates with 45–76 pooled animals per N unless otherwise noted. Data are mean ± SEM; * denotes p ≤ 0.05.

We next sought to determine the role of ALPK2 *in vivo* by depleting Alpk2 in zebrafish using splice-blocking morpholino oligonucleotides (MO). Injection of Alpk2 MOs caused alternative splicing and exon 4 removal to create a premature stop codon in exon 6 (Figures 2A–2D). Alpk2 morphants showed no observable structural defects at 24 hpf when compared with animals injected with scrambled negative control MOs (Figures 2E and 2F). However, transcriptional profiling of a panel of genes with known roles in early heart development (*nkx2.5, gata4, mef2ca, mef2cb, cmlc2, tnnt2, atp2a2, cacnalc2, cx43,* and *ryr2)* revealed that Alpk2 morphants had significantly reduced mRNA expression in all transcripts except *nkx2.5* and *ryr2* at 24 hpf (Figure 2G). At 48 hpf, Alpk2 morphants showed a significantly reduced heart beating rate (control 120.0 ± 1.29 bpm; alpk2 MO2 60.0 ± 2.19 bpm; Figure 2H) and cardiomyocyte numbers (*cmlc2:DsRed-nuc;* control 259 ± 21, *Alpk2 MO* 119 ± 8; 51 hpf; Figures 2I–2K). Of note, no difference in staining of endothelial cells was observed on the basis of activated leukocyte cell adhesion molecule (ALCAM; Figures 2I–2K). By 72 hpf, Alpk2 morphants displayed pericardial and yolk sac effusion and pronounced cardiac malformation coupled with defects in posterior development (Figures 2L–2O). We also assessed epicardium formation in fish lacking Alpk2 using zebrafish carrying a florescence epicardial cell reporter (*tcf21:DsRed* (Kikuchi et al., 2011). The zebrafish epicardium is the serosal epithelial lining of the heart derived from an extra-cardiac source of cells termed the proepicardium that is located adjacent to the pericardial sac and the venus pole during primitive heart tube formation (Plavicki et al., 2014, Serluca, 2008) and requires normal cardiac fitness to form (Peralta et al., 2013, Plavicki et al., 2014). In contrast to controls, Alpk2 morphants lacked *tcf21*^*+*^ ventricular epicardium at 96 hpf. During normal development, this is a time when the epicardium envelops the zebrafish heart ventricle but not the atrium (Figures 2P and 2Q; white arrowheads) (Plavicki et al., 2014).

**Figure 3:**
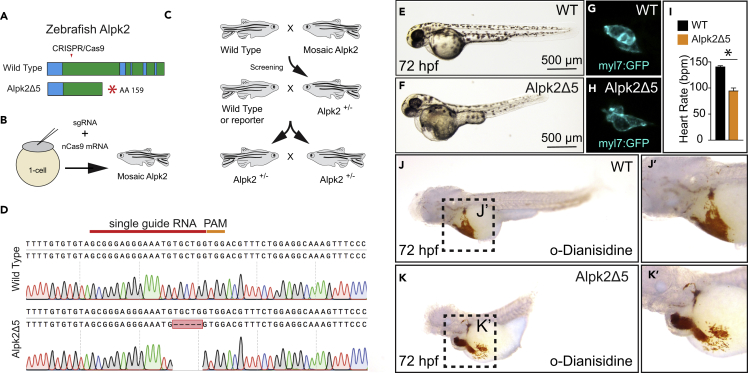
CRISPR/Cas9 Knockout of Zebrafish Alpk2 Inhibits Cardiac Development (A) Schematic of the zebrafish Alpk2 locus and CRISPR/Cas9 targeted region. * denote stop codon. (B and C) Injection and breeding paradigm to generate Alpk2 null homozygosity. (D–H) (D) Sanger sequencing of homozygous mutation showing 5-bp deletion. Representative bright-field (E, F; 72 hpf) and fluorescent (G, H; 120 hpf) images of wild-type (E, G) and Alpk2 null zebrafish (F, H) carrying a transgene for *myl7:GFP* (G, H). (I) Quantification of cardiac beating rate comparing Alpk2 null zebrafish to wild-type siblings at 72 hpf. * denotes p≤0.05. (J and K) Representative bright-field images of hemoglobin-peroxidase staining with *o*-Dianisidine in 72 hpf WT and homozygous Alpk2Δ5 embryos. Data are representative of N ≥ 3 independent breeding experiments consisting of n∼30–150 embryos per spawn. hpf, hours post fertilization.

There remains controversy regarding the use of MOs to study gene function (Rossi et al., 2015, Schulte-Merker and Stainier, 2014). To address this, we used CRISPR/Cas9 mutagenesis to induce indel mutations in exon 2 of Alpk2. Pilot studies indicated that the F0 fish had significant cardiac defects and posterior developmental anomalies that phenocopied the MO experiments. To avoid possible artifacts associated with mosaic mutations in the injected embryos, we derived an F1 line of fish with a heterozygous 5-base pair frameshift mutation within exon 2 of Alpk2 that resulted in a premature stop codon at amino acid 159 (Alpk2Δ5, Figures 3A–3D). Heterozygous fish showed no obvious developmental phenotype and were crossed to generate F2 homozygous Alpk2Δ5 fish. As with the Alpk2 morphants, there was no overt phenotype observed at 24 hpf in homozygous Alpk2Δ5 mutants. However, following heart tube formation, Alpk2Δ5 homozygotes display pericardial and yolk sac effusion, pronounced cardiac malformation coupled with reduced heart beating rate (Figures 3E–3I). Of note, cardiomyocyte numbers were not assessed in Alpk2Δ5 fish. Hemoglobin-peroxidase staining with *o*-Dianisidine shows that Alpk2Δ5 fish form erythrocytes but they lack blood circulation in the caudal trunk (Figures 3J–3K′). This suggests circulation defects that are secondary to reduced heart function. These results indicate that Alpk2 plays an important role in determining the number of cardiomyocytes, normal cardiac morphology, epicardium formation, and cardiac function.

### ALPK2 Promotes Differentiation of hESC-derived Cardiomyocytes

**Figure 4:**
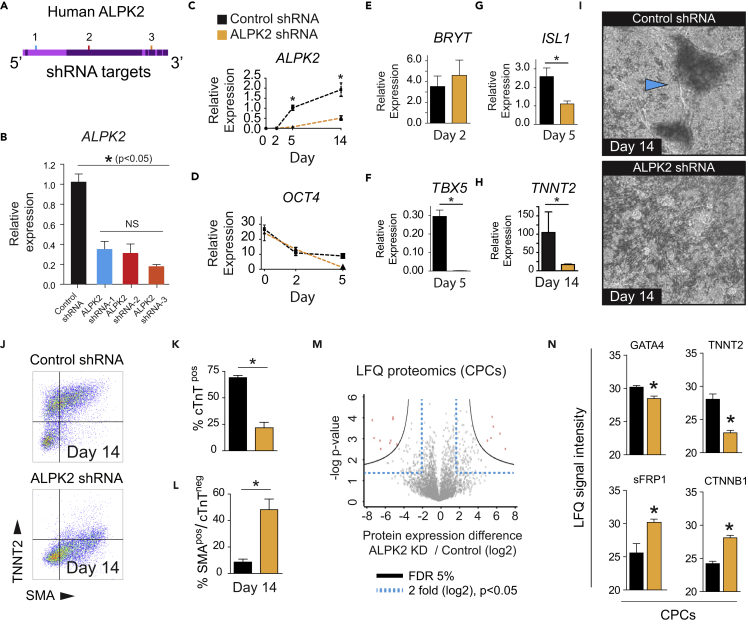
ALPK2 shRNA Knockdown Inhibits Cardiomyocyte Differentiation from hESCs (A) Schematic of ALPK2 locus and shRNA target sites. (B–D) (B) Validation of three ALPK2 shRNAs in human embryonic stem cell (hESC)-derived cardiomyocytes by qRT-PCR 3 days following viral transfection. Transcript expression analysis of *ALPK2* (C) and *OCT4* (D) following control or ALPK2 lentiviral shRNA transfection over a time course of cardiomyocyte differentiation. (E–H) RT-qPCR analysis of mesoderm (*BRYT*), primary (*TBX5*) and secondary (*ISL1*) heart field genes, and the cardiac structural gene (*TNNT2*). (I) Representative bright-field images of control and ALPK2 shRNA-treated cells following directed differentiation toward cardiomyocytes (day 14). Arrowhead denotes contractile cardiomyoyctes. (J–L) Flow cytometry (y axis, TNNT2; x axis, smooth muscle actin, SMA) on differentiation day 14 following control and ALPK2 shRNA lentiviral transduction (N = 3). (M) Volcano plot of comparing cardiac progenitor cells (CPCs) transfected with control or ALPK2 shRNAs by system-wide label-free quantitative proteomics (FDR, false discovery rate). (N) Protein expression of TNNT2, GATA4, sFRP1, and CTNNB1 (LFQ signal intensity; log2 transformed) in control and ALPK2 shRNA CPCs derived from hESCs. Proteomic data are representative of two biological replicates that were pooled, processed, and measured in triplicate. Data are mean ± SEM; * denotes p ≤ 0.05; LFQ, label-free quantification. See also Figures S2 and S3.

To determine whether ALPK2 plays a functional role in human cardiomyocyte development, ALPK2 was knocked down (KD) by lentiviral short hairpin RNAs (shRNA) in hESCs (Figures 4A and 4B), and cells were subjected to cardiomyocyte differentiation. ALPK2 KD significantly diminished ALPK2 expression throughout the differentiation process (Figure 4C) without disrupting hESC colony morphology or pluripotency-associated gene expression (Figure 4D). To assess the role of ALPK2 during early developmental stages, transcript abundance of markers of mesoderm, CPCs, and definitive cardiomyocytes were assessed on days 2, 5, and 14 of differentiation (Figure 1D). Although there was no change in the expression of the pan-mesoderm marker, *BRYT*, expression of CPC markers, *TBX5* and Islet 1 (*ISL1*), and *TNNT2* was downregulated in ALPK2 KD cells (Figures 4E–4H). In addition, ALPK2 KD cells preferentially differentiated toward a *TNNT2-*negative and smooth muscle actin (SMA)-positive cell type (Figures 4I–4L). These data suggest ALPK2 plays a role during transitioning from mesoderm toward the cardiomyocyte lineage. To further test this hypothesis, we assessed the differentiation efficiency of ALPK2 KD in forming endocardial-like EC and hemogenic EC from mesodermal sub-populations. These results showed no effect on the percentage of endocardial-like or hemogenic ECs (based on KDR/CD34 expression on day 5 of differentiation, Figure S2), indicating ALPK2 specifically plays a role in potentiating the cardiomyocyte lineage.

We characterized potential downstream effectors of ALPK2 by performing label-free quantitative (LFQ) proteomics in hESCs undergoing cardiac-directed differentiation (Figure S3A, Table S2: All proteins identified by LFQ proteomics. Related to Figure 4). To assess the effect on early cardiac cell fate specification, hESCs were transduced with either control or ALPK2 shRNAs and analyzed as hESCs or after differentiation as CPCs. Normalized protein digests were measured by nano-liquid chromatography-tandem mass spectrometry (LC-MS/MS) on a Fusion Orbitrap mass spectrometer and quantified using label-free methods (Hofsteen et al., 2016). Label-free quantification was reproducible across all samples, with an average Pearson's correlation of 0.90 (Figure S3B; representative image). To validate our approach, we analyzed TNNT2 and ALPK2 protein expression over hESC cardiomyocyte differentiation. As expected, both TNNT2 and ALPK2 protein levels increased as cells differentiated toward cardiomyocytes (Figures S3C and S3D). ALPK2 protein expression increased progressively as control hESCs differentiated into CPCs and then into cardiomyocytes, paralleling the mRNA expression pattern. Following treatment with ALPK2 shRNA lentiviruses, ALPK2 protein was decreased in CPCs and cardiomyocytes (Figure S3D).

Next, we performed unbiased hierarchal cluster analysis of samples and found replicates and experimental conditions cluster together (Figure S3E). We analyzed ALPK2-regulated proteins by using p ≤ 0.05 and a cutoff of 2-fold (log2) change. This analysis identified 241 ALPK2-regulated proteins in CPCs (126 upregulated, 115 downregulated; Figure 4M, Table S3: ALPK2-regulated proteins identified by LFQ proteomics in cardiac progenitor cells. Related to Figure 4). We observed no ALPK2-regulated proteins between control and ALPK2 KD undifferentiated hESCs. Gene ontology (GO) term analysis revealed that ALPK2 regulates processes involved in RNA and protein binding, protein kinase binding, cell-cell adhesion, the extracellular matrix, and actin filaments and function, and canonical WNT signaling (Tables S4: GO Terms enriched by ALPK2 knockdown identified by LFQ proteomics in cardiac progenitor cells. Related to Figure 4, Table S5: GO Terms repressed by ALPK2 knockdown identified by LFQ proteomics in cardiac progenitor cells. Related to Figure 4). Specifically, ALPK2 KD reduced TNNT2 and GATA4 expression in CPCs (Figure 4N) and induced the expression of the WNT/β-catenin antagonist and secreted frizzled-related protein (sFRP1) (Ren et al., 2013) as well as the WNT signaling transducer β-catenin (CTNNB1) (Figure 4N). These proteomic data suggest ALPK2 regulates key mediators of the WNT/β-catenin signaling pathway and cardiac developmental proteins during cardiomyocyte differentiation.

### ALPK2 Negatively Regulates WNT/β-Catenin for Cardiac Development

**Figure 5:**
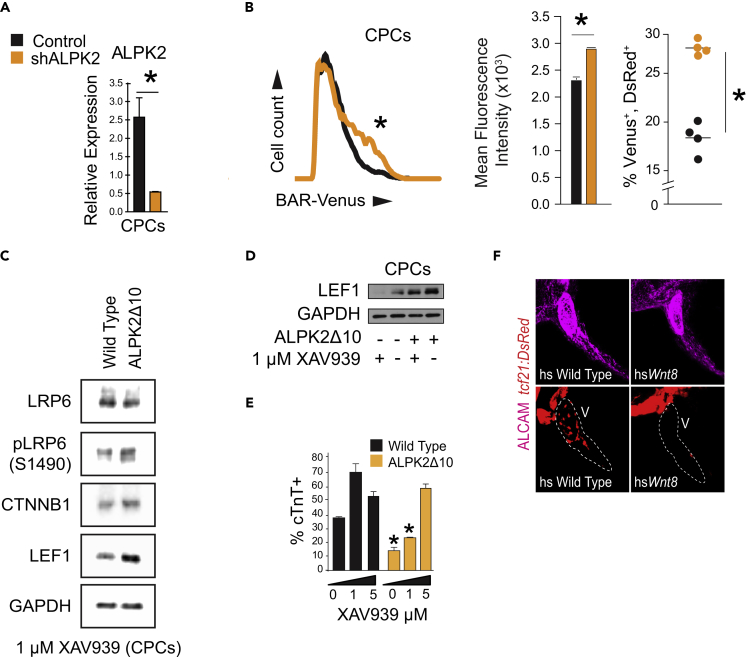
ALPK2 Inhibits WNT/β-Catenin Signaling to Promote Cardiogenesis (A) Transcripts abundance of *ALPK2* in control and ALPK2 shRNA-transfected cardiac progenitor cells (CPCs) derived from human embryonic stem cells (hESCs). (B) Flow cytometric plot and graphs quantifying venus expression driven by β-catenin activated reporter (BAR) in live CPCs following control or ALPK2 shRNA transfection as hESCs. (C) Western blot protein expression analysis of WNT/β-catenin signaling modulators in wild-type and CRISPR/Cas9-mediated ALPK2 mutant hESC-derived CPCs (ALPK2Δ10) treated with a small molecule direct tankyrase inhibitor XAV939 (1 μM). (D) Western blotting for the WNT/β-catenin signaling target LEF1 in wild-type and ALPK2Δ10 cells with or without the addition of XAV939 (0 or 1 μM). (E) TNNT2 flow cytometry quantification of day 14 cardiomyocytes derived from wild-type and ALPK2Δ10 hESCs treated with XAV939 (0, 1, 5 μM). (F) Representative images of heat shocked (hs) wild-type and hsWnt8b transgenic zebrafish carrying a transgene for the epicardium (*tcf21:DsRed*) at 96 hpf. Fish were counterstained with an activated leukocyte cell adhesion molecule (ALCAM; magenta). N = 3–5 biological replicates, and data are displayed as mean ± SEM. * = p ≤ 0.05. See also Figure S4.

The LFQ proteomics indicate that ALPK2 knockdown affects WNT/β-catenin signaling during cardiac development. To more definitively assess the role of ALPK2 in regulating WNT/β-catenin signaling, we stably transduced a lentiviral BAR driving venus expression in hESCs (Figure S4A). BAR-hESCs were subsequently transduced with control or ALPK2 shRNAs and were subjected to directed differentiation toward CPCs, followed by assessment of venus expression analysis by live-cell flow cytometry (Figure S4B). ALPK2 KD significantly reduced *ALPK2* expression (Figure 5A) and increased the overall mean live-cell venus fluorescence intensity (geometric mean: control, 2.39 ± 0.03 x 10^3^; ALPK2 KD, 3.01 ± 0.44 x 10^3^) and significantly increased the percentage of venus-expressing CPCs (venus^+^/DsRed^+^; control %, 18.4 ± 0.8; ALPK2 KD %, 27.6 ± 0.4) (Figure 5B), indicating ALPK2 KD increases β-catenin-dependent WNT signaling.

To further assess the role of ALPK2 and WNT/β-catenin signaling we used CRISPR/Cas9 gene editing to mutate the protein kinase domain of ALPK2 in hESCs. This approach successfully created an hESC line carrying a homozygous 10-bp frameshift deletion causing a premature stop codon in exon 9 (ALPK2Δ10; Figures S4C and S4D). Wild-type and ALPK2Δ10 hESCs were differentiated to CPCs (Figure 1D), and transcript abundance of *ALPK2* was measured by RT-qPCR. ALPK2Δ10 CPCs had a significantly reduced *ALPK2* expression when compared with control (Figure S4E), suggesting ALPK2Δ10 cells undergo nonsense-mediated mRNA decay. ALPK2Δ10 and wild-type hESCs were subsequently differentiated toward cardiomyocytes as described in Figure 1D and analyzed by immunoblotting and TNNT2 flow cytometry on days 5 and 14, respectively. We characterized the expression of LRP6, pLRP6, CTNNB1, and LEF1 as indicators of active WNT/β-catenin signaling in CPCs lacking ALPK2. The WNT co-receptor, LRP6, is phosphorylated by glycogen synthase kinase 3 beta (GSK3β) and casein kinase 1 (CK1) upon WNT ligand binding (Davidson et al., 2005, Zeng et al., 2005). Furthermore, induction of WNT/β-catenin signaling is associated with induced expression of target proteins such as LEF1 (Hsu et al., 1998). The expression of pLRP6, CTNNB1, and LEF1 proteins was increased in ALPK2Δ10 CPCs when compared with wild-type CPCs (Figure 5C). To further examine the role of ALPK2 during WNT/β-catenin signaling we analyzed the expression of LEF1 using wild-type and ALPK2Δ10 CPCs with or without a small molecule WNT/β-catenin signaling inhibitor, XAV939 (0 or 1 μM). In wild-type cells, XAV939 reduced LEF1 expression to almost undetectable levels. Although baseline LEF1 levels are increased in ALPK2Δ10 CPCs, treatment with XAV939 also reduced LEF1 protein abundance (Figure 5D), suggesting that at least part of the ALPK2's Wnt-suppression effect occurs upstream of the Axin-stabilizing enzyme, tankyrase, the target for XAV939 (Huang et al., 2009).

Next, we sought to rescue the ALPK2-mediated cardiomyocyte differentiation defects by attenuating WNT/β-catenin signaling with increasing doses of the pathway inhibitor, XAV939. Wild-type and ALPK2Δ10 cells were exposed to 0, 1, and 5 μM XAV939 on day 3 of the differentiation protocol for 2 days, and cells were collected on day 14 for flow cytometry analysis (TNNT2). XAV939 showed a dose-dependent ability to restore the cardiogenic potential of ALPK2Δ10 cells, such that exposure to 5 μM XAV939 restored cardiac differentiation to wild-type levels (Figure 5E). Thus inhibition of WNT/β-catenin signaling can restore cardiomyocyte differentiation deficiencies observed in ALPK2Δ10 cells.

WNT/β-catenin signaling pathway modulation is required for the differentiation of the epicardium in hESCs (Bao et al., 2016, Iyer et al., 2015), and Alpk2-depleted zebrafish showed impaired formation of the epicardium (Figure 2). To test more specifically for a role of WNT/β-catenin signaling in epicardium formation in zebrafish, we genetically overexpressed *wnt8* to induce canonical WNT signaling using a heat-shock-inducible zebrafish line (*hsp70:wnt8b-GFP* [Weidinger et al., 2005]) crossed to an epicardial cell reporter (*tcf21:DsRed*). Fish were subjected to heat shock following heart tube formation (24 and 48 hpf), and epicardial formation was assessed at 96 hpf. As with zebrafish depleted of Alpk2, late embryo-larval activation of WNT/β-catenin signaling inhibited the formation of the epicardium (Figure 5F). Collectively, these data demonstrate that inhibition of WNT/β-catenin signaling by ALPK2 is required for the formation of both the myocardium and epicardium.

### Quantitative Phosphoproteomics Reveals Phosphorylation Motif and Candidate Substrate Proteins for ALPK2

**Figure 6:**
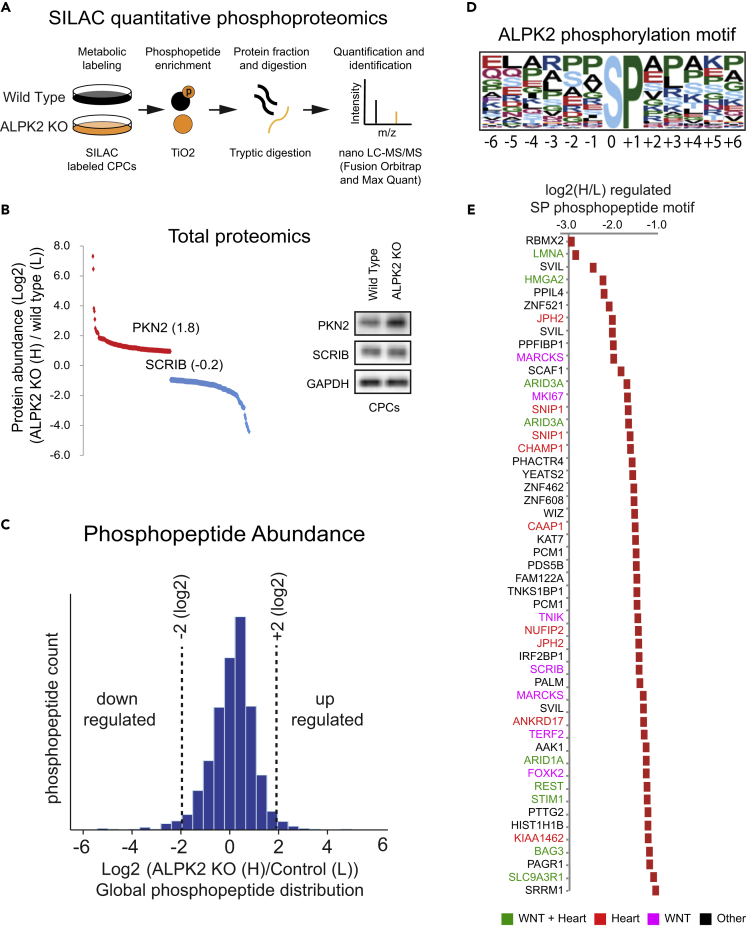
Quantitative Phosphoproteomics Reveals Phosphorylation Motif and Candidate Substrate Proteins for ALPK2 (A) Schematic of stable isotype labeling of amino acids in cell culture (SILAC) quantitative phosphoproteomics using wild-type and ALPK2Δ10 cardiac progenitors differentiated from human embryonic stem cells (hESCs). (B, left) Waterfall plot of differential protein abundance comparing wild-type and ALPK2Δ10 CPCs. (B, right) Western blot for Protein Kinase N2 (PKN2) and Scribble (SCRIB), N = 3 independent differentiations. (C) Total distribution of phosphopeptide abundance comparing wild-type and ALPK2Δ10 CPCs. (D and E) Putative ALPK2 phosphorylation substrate motif and (E) proteins lacking phosphorylation events as a result of ALPK2 mutation. Proteins are color coded indicating their role(s) in both WNT signaling and heart development or disease (green), heart development or disease (red), WNT only (magenta), or other (black). See also Figure S5.

To identify ALPK2 targets we conducted quantitative phosphoproteomics comparing wild-type with ALPK2Δ10 CPCs (see Transparent Methods in Supplemental Information and Figure 6A). Wild-type and ALPK2Δ10 CPCs were collected and processed for proteomics and phosphoproteomics following stable isotope labeling with amino acids in cell culture (SILAC) and directed differentiation. We detected 2,664 proteins, of which 317 (157 induced, 160 repressed) were differentially expressed (≥2-fold; log2; Figures 6B and S5, and Table S6: All proteins identified by SILAC proteomics. Related to Figure 6). GO term analysis revealed that ALPK2-regulated proteins have known roles in negative regulation of WNT/β-catenin signaling and β-catenin-TCF complex assembly along with proteins that play roles in regulating heart development and actin behavior and function (Figure S6C, Table S7: GO Terms enriched in ALPK2Δ10 cardiac progenitor cells identified by SILAC proteomics. Related to Figures 6 and S8: GO Terms repressed in ALPK2Δ10 cardiac progenitor cells identified by SILAC proteomics. Related to Figure 6).

We validated the proteomics screen by immunoblotting for two proteins with known roles in modulating canonical and non-canonical WNT signaling. Protein kinase N2 (PKN2) has previously been described as a negative regulator of WNT/β-catenin signaling (James et al., 2013) and was predicted to be upregulated in CPCs derived from ALPK2Δ10 hESCs. Alternatively, the cell polarity and non-canonical WNT signaling regulator, Scribble (SCRIB) (Sisson et al., 2015), was predicted to be unchanged in ALPK2Δ10 CPCs. Western blotting for PKN2 and SCRIB confirmed that in ALPK2Δ10 CPCs, PKN2 was upregulated and SCRIB was not changed (Figure 6B).

We also detected and quantified 1,972 phosphopeptides corresponding to 846 proteins (Figure 6C, Table S9: All phosphopeptides identified and differentially phosphorylated in ALPK2Δ10 cardiac progenitor cells identified by SILAC proteomics. Related to Figure 6). Phosphorylation motif analysis revealed that ALPK2 is predicted to target proteins carrying serine-proline amino acid residues (Figure 6D). Of the predicted ALPK2 target substrates (proteins that lack phosphorylation in ALPK2Δ10 CPCs), approximately one-third have known roles in regulating WNT/β-catenin signaling (32.6%) and heart development and disease (34.7%, Figure 6E). Thus our data support the hypothesis that ALPK2 regulates cardiac development by phosphorylating substrates that are associated with WNT/β-catenin signaling during heart development.

## Discussion

Regulation of WNT/β-catenin signaling has long been known to be essential for heart development, homeostasis, and disease (Hofsteen et al., 2016, Moon et al., 2004, Moon et al., 2017, Ozhan and Weidinger, 2015, Ueno et al., 2007). We used targeted combinatorial screening from cardiomyocyte differentiation of hESCs coupled with siRNA screen of human cancer cells to identify previously unknown regulators of heart development and WNT/β-catenin signaling. In this study, we demonstrate that knockdown and knockout of ALPK2 *in vivo* and *in vitro* result in diminished cardiogenesis and increased WNT/β-catenin signaling activity. ALPK2 is highly expressed in a stage-dependent manner during cardiogenesis and is an important regulator of cardiomyocyte differentiation in hESCs and cardiac development in zebrafish. Furthermore, we find that ALPK2 regulates cardiogenesis by repressing WNT/β-catenin signaling following cardiac commitment. We sought to understand how ALPK2 regulates WNT/β-catenin signaling during cardiogenesis by performing quantitative proteomic screens. These screens further supported our combinatorial efforts by identifying that ALPK2 regulates cardiac proteins along with mediators of the WNT/β-catenin signaling pathway. We consistently observe an increased expression of proteins that inhibit WNT/β-catenin signaling following depletion of ALPK2. Key examples of this are the canonical WNT proteins, PKN2, and sFRP1 that could very likely be induced as a compensatory mechanism to repress the pathway.

Comparing multiple model systems to understand gene function is critical owing to gene duplication, species variation, or compensatory mechanisms (Jensen et al., 2013, Olson, 2006, Rossi et al., 2015). Expression analyses of *alpk2* in zebrafish embryos demonstrate that it is expressed in the developing heart, whereas in human ESCs, *ALPK2* is expressed in the cardiac progenitor cells and cardiomyocytes. Correspondingly, both model systems show significant defects in cardiogenesis when ALPK2 is knocked down or knocked out.

Taken together, the vertebrate heart requires precise control of signaling cascades to orchestrate dynamic cell fate decisions and morphogenetic changes during development. We identified that the regulation of the WNT/β-catenin signaling pathway by ALPK2 plays a critical role in hESC-CM differentiation and in cardiac maturation in zebrafish. Thus we report ALPK2 is an evolutionarily conserved alpha protein kinase that is important for the earliest stages of vertebrate heart development through repression of WNT/β-catenin signaling.

## Methods

All methods can be found in the accompanying Transparent Methods supplemental file.

## Supplemental Information

Document S1. Transparent Methods and Figures S1–S5

Table S1. Human and Zebrafish Oligonucleotides Using for Gene Targeting and RT-qPCR, Related to Figures 1, 2, 3, 4, and 5

Table S2. All Proteins Identified by Label-free Quantitative (LFQ) Proteomics, Related to Figure 4

Table S3. ALPK2 Regulated Proteins Identified by Label-free Quantitative (LFQ) Proteomics in Cardiac Progenitor Cells, Related to Figure 4

Table S4. GO Terms Enriched by ALPK2 Knockdown Identified by Label-free Quantitative (LFQ) Proteomics in Cardiac Progenitor Cells, Related to Figure 4

Table S5. GO Terms Repressed by ALPK2 Knockdown Identified by Label-free Quantitative (LFQ) Proteomics in Cardiac Progenitor Cells, Related to Figure 4

Table S6. All Proteins Identified by Stable Isotope Labeling of Amino Acids in Cell culture (SILAC) Proteomics, Related to Figure 6

Table S7. GO Terms Enriched in ALPK2Δ10 Cardiac Progenitor Cells Identified by Stable Isotope Labeling of Amino Acids in Cell Culture (SILAC) Proteomics, Related to Figure 6

Table S8. GO Terms Repressed in ALPK2Δ10 Cardiac Progenitor Cells Identified by Stable Isotope Labeling of Amino Acids in Cell Culture (SILAC) Proteomics, Related to Figure 6

Table S9. All Phosphopeptides Identified and Differentially Phosphorylated in ALPK2Δ10 Cardiac Progenitor Cells Identified by Stable Isotope Labeling of Amino Acids in Cell Culture (SILAC) Proteomics, Related to Figure 6
