# Supplementary material for: ALPK2 Promotes Cardiogenesis in Zebrafish and Human Pluripotent Stem Cells
Source: iScience. 2018 Apr 9;2:88–100. doi: 10.1016/j.isci.2018.03.010 (PMC5993047; doi:10.1016/j.isci.2018.03.010)
Supplement: Document S1. Transparent Methods and Figures S1–S5 [file mmc1.pdf]

ISCI, Volume *2*

Supplemental Information

ALPK2 Promotes Cardiogenesis in Zebrafish and Human Pluripotent Stem Cells

Peter Hofsteen, Aaron Mark Robitaille, Nicholas Strash, Nathan Palpant, Randall T. Moon, Lil Pabon, and Charles E. Murry

Transparent Methods

Cell culture and cardiac directed differentiation.

Human embryonic stem cells (RUES2) were maintained and cardiac directed differentiation was performed based on methods previously described (Palpant et al., 2015a). Briefly, hESCs were seeded on Matrigel (BD) coated plates and directed differentiation was performed using a combination of activin A and BMP4 and small molecule activation and inhibition of the WNT/β-catenin signaling pathway as illustrated in Figure 1D. Endothelial differentiation was performed as previously described (Palpant et al., 2015b). Briefly, differentiation was initiated with Activin A (100 ng/mL for endocardial-like endothelium (EECs) and 50 ng/mL for hemogenic endothelium (HECs)) in RPMI B27 without insulin and 1x matrigel. On day 1, media was changed to BMP4 (5 ng/mL for EECs and 40 ng/mL for HECs) in RPMI B27 minus insulin with 1 μM CHIR-99021. On day 2 media was changed to Stempro34 (Invitrogen, 10640019) backbone media containing 200 ng/mL VEGF (Peprotech, 100-20), 5 ng/mL bFGF (Peprotech, 100-18B), 10 ng/mL BMP4 (R&D SYSTEMS, 314-BP-050), 4x10-4 M monothioglycerol, 50 μg/mL Ascorbic Acid, 2mM L-Glutamine (Invitrogen, 25030-081), and pen-strep (Invitrogen, 15140-163) and remained until day 5 when cells were phenotyped.

Zebrafish strains and husbandry.

Wild-type (AB; zebrafish International Resource Center, Eugene, OR, USA), *tcf21:DsRed2* (Kikuchi et al., 2011), *cmlc2:DsRed-nuc* (Mably et al., 2003), *myl7:GFP* (Burns et al., 2005) and *hsWNT8:GFP* (Weidinger et al., 2005) were used and maintained using standard procedures (Westerfield, 2000) in accordance with the Institutional Animal Care and Use Committee-approved protocols.

Flow Cytometry.

Cardiomyocyte purity and immaturity was assessed by flow cytometry using cardiac troponin T (Thermo Scientific, 1:100) antibody and smooth muscle actin (SMA; Abcam, 1:200) or the isotype control on day 14 of hESC directed differentiation. Human ESC derived endothelial cells were analyzed on day 5 with antibodies against human VEGF

R2/KDR PE (R&D Systems) and CD34 (BD Biosciences, 340430). Cells were analyzed using a BD FACSCANTO II or sorted on a BD FACSARIA II (Beckton Dickinson, San Jose, CA) with FACS Diva software (BD Biosciences). Instrument settings were adjusted to avoid spectral overlap.

Quantitative Reverse Transcriptase PCR (RT-qPCR).

Total RNA isolation was performed as per manufacturers' protocol (RNeasy Miniprep Kit, QIAGEN). First-strand cDNA from 500 ng total RNA was synthesized using the Superscript III enzyme kit (Invitrogen). Quantitative RT-PCR was conducted using the Sensimix SYBR PCR Kit (Bioline) on a 7900HT Fast-Real-Time PCR system (Applied Biosystems). Relative transcript values were normalized to either HPRT (human) or β-actin (zebrafish) and primers are listed in Table S1.

Analysis of RNA-seq and chromatin dynamics.

Figure 1C is based on the following data set: NCBI Gene Expression Omnibus (GEO; GSE35583, epigenetics; GSE19090, gene expression; previously deposited (Paige et al., 2012). Figure 1F is based on the following data set: GEO: GSE97080 (Palpant et al., 2017).

In situ hybridization.

Wild type zebrafish embryos were collected at 13 and 22 hpf, fixed in 4% paraformaldehyde, dehydrated to methanol, and stored at -20°C until needed. A 613 base pair fragment spanning of *alpk2* was amplified from embryonic zebrafish cDNA using primers listed in Table S1 and the digoxigenin-UTP (Roche) antisense *alpk2* riboprobe was synthesized using T7 RNA polymerase (Promega). The *gata4* riboprobe was previously synthesized (Paige et al., 2012) and *in situ* hybridization was performed as previously described (Hofsteen et al., 2013). Images were obtained using a Nikon SMZ1500 microscope using a Nikon Digital DS-Ri1 camera.

Morpholino Oligonucleotides.

Translation and splice blocking morpholino oligonucleotides (MO) (GeneTools LLC,

Philomath, OR) were injected into one-cell zebrafish embryos (0.25-1 ng/embryo). Validation of splicing was confirmed by reverse transcriptase PCR following first strand cDNA synthesis (Invitrogen) using primers designed to amplify the flanking region of intron-exon 4 (f1, r1; exons 4-6) as well as downstream the splice junction (f2, r2; exons 5-6). Primers and MO sequences can be found in Table S1 and validation is illustrated in Figure 2A-D.

CRISPR/Cas9 gene editing

Human and zebrafish guide sequences were designed using CRISPRscan software (Moreno-Mateos et al., 2015) and are listed in Table S1. Human sgRNA oligonucleotides were designed with overhangs for ligation into a BbsI (ThermoFisher Scientific) digested pSpCas9(BB)-2A-Puro (px459) V2.0 (gifted from Feng Zhang, Addgene plasmid # 62988) single vector expression system (Ran et al., 2013). Human PSCs were transfected with 1 µg of the ALPK2 sgRNA-PX459 plasmid using GeneJuice (EMD Millipore and hESCs with plasmid integration were selected using puromycin (InvivoGen). Confirmation of mutation and stop codon was conducted by Sanger sequencing. Zebrafish sgRNA and nCas9 was synthesized, injected, and screed as previously described (Hofsteen et al., 2016). Founder fish (F0) were outcrossed to with wild type AB fish to generate a stable Alpk2 hemizygous line (F1). F1 fish were subsequently propagated and hemizygous fish were bred to attain homozygosity. Validation of mutations were conducted through Sanger sequencing. Guide sequences and primers used for validation are listed in Table S1.

Immunohistochemistry and confocal microscopy.

Zebrafish antibody staining was performed as previously described (Plavicki et al., 2013). Primary antibody used was rabbit anti-DsRed (AnaSpec, Fremont, CA) at 1:200 in phosphate buffered saline with 0.03% triton and 4% bovine serum albumin (PBT) followed by secondary antibody Alexafluor 568 at 1:100 in PBT. Samples were mounted with Vectashield containing DAPI (Vector Laboratories) and images were obtained using a Nikon A1R confocal mounted on a Nikon TiE inverted microscope.

***Zebrafish heart rate, cardiomyocyte quantification*, and O-dianisidine staining.**

Heart rate was collected by counting ventricular contractions from randomly selected fish over a period of one minute at 27˚C (Video S1). Zebrafish (*cmlc2:DsRed-nuc*) were injected with control or *Alpk2* MO, fixed at 48 hpf and processed for IHC as described above. Fish were stained for rabbit anti-DsRed (1:200, AnaSpec, Fremont, CA) and mouse anti-activated leukocyte cell adhesion molecule (ALCAM, 1:50) primary antibodies followed by secondary staining with Alexaflour-596 anti-rabbit and -633 anti-mouse at 1:100. Hearts were imaged (z-series) by confocal microscopy and cardiomyocytes were counted in a blinded fashion. O-dianisidine staining was performed by staining control and Alpk2Δ5 zebrafish at 72 hpf with a o-dianisidine solution (o-dianisidine (0.6 mg/ml), 0.01 M sodium acetate (pH 4.5), 0.65% H202, and 40% (vol/vol) ethanol)) as previously described (Paffett-Lugassy and Zon, 2005). Briefly, fish were dechorionated and stained in the dark for 25 minutes at room temperature, washed 3x with deionized H20 and fixed overnight with 4% paraformaldehyde in PBS (vol/vol) at 4˚C. Embryos were bleached as described (Paffett-Lugassy and Zon, 2005) and imaged using a Nikon SMZ1500 microscope using a Nikon Digital DS-Ri1 camera.

***Short hairpin RNA (shRNA) and lentiviral transduction*.**

Three non-overlapping shRNAs targeting various regions of the ALPK2 transcript were inserted into a PLKO.1 vector containing a puromyocin resistance cassette (TRCN0000230560, NM_05947.3-368s21c1; TRCN0000230561, NM_05947.3-1217s21c1; TCRN0000230562, NM_052947.3-5858s21c1; Sigma, Table S1). Lentivirus was synthesized and cells were transfected in suspension containing 10 μM Y-27632 (Sigma). For control, cells were transduced with a lentivirus containing a non-targeting shRNA (Sigma). Cells were transduced for 48 hours, puromyocin selected and passaged for expansion once prior to cardiac differentiation. To validate the efficiency of ALPK2 knockdown, we transduced cardiomyocytes with three non-overlapping ALPK2 shRNAs. All ALPK2 shRNAs resulted in significant reduction (72-83%) of ALPK2 when compared to the empty vector shRNA controls (Figure 4B). For assessing WNT/β-catenin activity, hESCs were transduced with a lentiviral β-catenin-activated reporter (BAR) driving venus as previously reported (Palpant et al., 2015b).

Western Blotting

Cells were lysed in ice-cold cell lysis buffer (Cell Signaling, cat# 9803) containing protease inhibitors (Calbiochem, cat# 539134) and quantification of total protein was conducted using the Pierce BCA Protein Assay Kit (Thermo Scientific). Normalized total protein was subjected to SDS-PAGE and western blotting was performed as previously described (Yang et al., 2014). Primary antibodies used are listed below were coupled with either anti-mouse, anti-goat or anti rabbit horseradish peroxidase secondary antibody at 1:5000 (Santa Cruz Biotechnology).

	Manufacturer	Host	Dilution	Lot
CTNNB1	Cell Signaling	Rabbit	1 / 1000	9562S
LRP6	Cell Signaling	Rabbit	1 / 1000	3395
phospho-LRP6 (S1490)	Cell Signaling	Rabbit	1 / 1000	2568
LEF1	Cell Signaling	Rabbit	1 / 1000	2230
PKN2	Bethyl Labs	Rabbit	1 / 1000	A302-443A
SCRIB	Santa Cruz	Goat	1 / 1000	sc-11049
GAPDH	Abcam	Mouse	1 / 5000	ab8245

Proteomics

Label Free Quantitative Proteomics was conducted as previously published (Hofsteen et al., 2016) and described in detail in the supplemental experimental procedures. In brief, hESCs, CPCs and cardiomyocytes treated with either control or ALPK2 shRNA (2 biological replicates per time point/treatment) were pooled and washed in 1xPBS and flash frozen. Cell pellets were lysed in 1M urea, 50mM ammonium bicarbonate, pH 7.8, and heated to 50°C for 20min. Cell debris was removed by centrifugation (1000g, 2min). Following a BCA assay, normalized quantities of protein were reduced with 2mM DTT, alkylated with 15mM iodoacetamide, and digested overnight with a 1:50 ratio of trypsin to total protein. The resulting peptides were desalted on Waters Sep-Pak C18 cartridges. Peptides were measured by nano-LC-MS/MS on a Thermo Scientific Fusion Orbitrap. Peptides were separated online by reverse phase chromatography using a heated 50°C 30cm C18 column (75mm ID packed with Magic C18 AQ 3μM /100A

beads) in a 180min gradient (1% to 45% acetonitrile with 0.1% formic acid) separated at 250nL/min. The Fusion Orbitrap was operated in data-dependent mode with the following settings: 60000 resolution, 400-1600 m/z full scan, Top Speed 3 seconds, and an 1.8 m/z isolation window. Identification and label free quantification (LFQ intensity) of peptides was done with MaxQuant 1.5 using a 1% false discovery rate (FDR) against the human Swiss-Prot/TrEMB database downloaded from Uniprot on October 11th, 2013. The databases contained forward and reverse human sequences as well as common contaminants. Peptides were searched using a 5 ppm mass error and a match between run window of 2 min. Proteins that were significantly regulated between conditions were identified using a permutation-based t-test (S1, FDR 5%) in Perseus

SILAC Proteomics: Undifferentiated RUES2 cells were metabolically labeled in conditioned media containing R0K0 (light) or R10K8 (heavy) SILAC DMEM/F-12 media (Dundee Cells Products) for 6 passages. Cells were then differentiated into cardiomyocytes in R0K0 (light) or R10K8 (heavy) RPMI SILAC media (Dundee Cells Products). Cells were washed in 1xPBS and flash frozen. Cell pellets were lysed in 1M urea, 50mM ammonium bicarbonate, pH 7.8, and heated to 50°C for 20min. Cell debris was removed by centrifugation (1000g, 2min). Following a BCA assay, normalized quantities of protein were reduced with 2mM DTT, alkylated with 15mM iodoacetamide, and digested overnight with a 1:50 ratio of trypsin to total protein. The resulting peptides were desalted on Waters Sep-Pak C18 cartridges. Phosphopeptides were then enriched using Pierce Titanium Dioxide Phosphopeptide Enrichment Kit (Thermo Fisher Scientific) according to the manufactures instructions. Peptides were measured by nano-LC-MS/MS on a Fusion Orbitrap (Thermo Fisher Scientific). Peptides were separated online by reverse phase chromatography using a heated 50°C 30cm C18 columns (75mm ID packed with Magic C18 AQ 3μM /100A beads) in a 180min gradient (1% to 45% acetonitrile with 0.1% formic acid) separated at 250nL/min. The Fusion was operated in data-dependent mode with the following settings: 60000 resolution, 400-1600 m/z full scan, Top Speed 3 seconds, and an 1.8 m/z isolation window. Identification and label free quantification of peptides was done with MaxQuant 1.5 using a 1% false discovery rate (FDR) against the human Swiss-Prot/TrEMB database

downloaded from Uniprot on June 2nd, 2016. The databases contained forward and reverse human sequences as well as common contaminants. We analyzed two biological replicates per condition with six technical replicates. Peptides were searched using a 5ppm mass error and a match between run window of 2min. Gene ontology term analysis was conducted using DAVID functional analysis.

Statistics. Single variable analysis between two samples was carried out using Student's t-test. Single and multivariable assays were analyzed by one- or two-way ANOVA. Results are presented as mean±SEM. For all statistically significant results, *P<0.05.

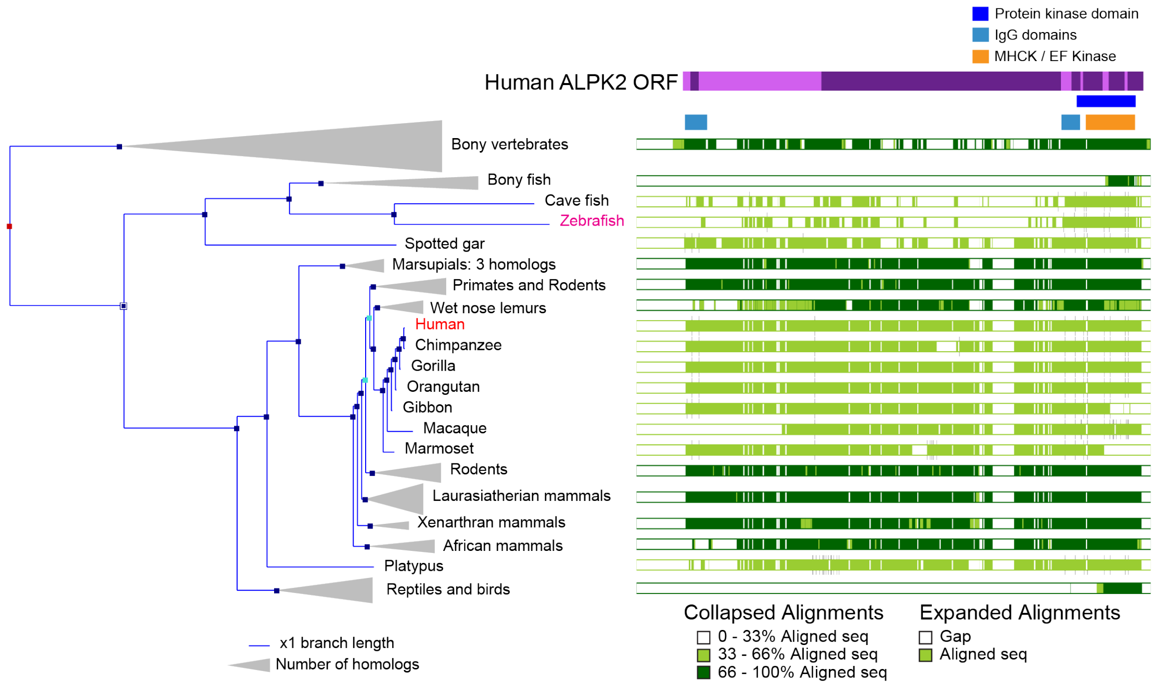

Figure S1: ALPK2 conservation and sequence alignment modified from ensembl. Related to figure 1.

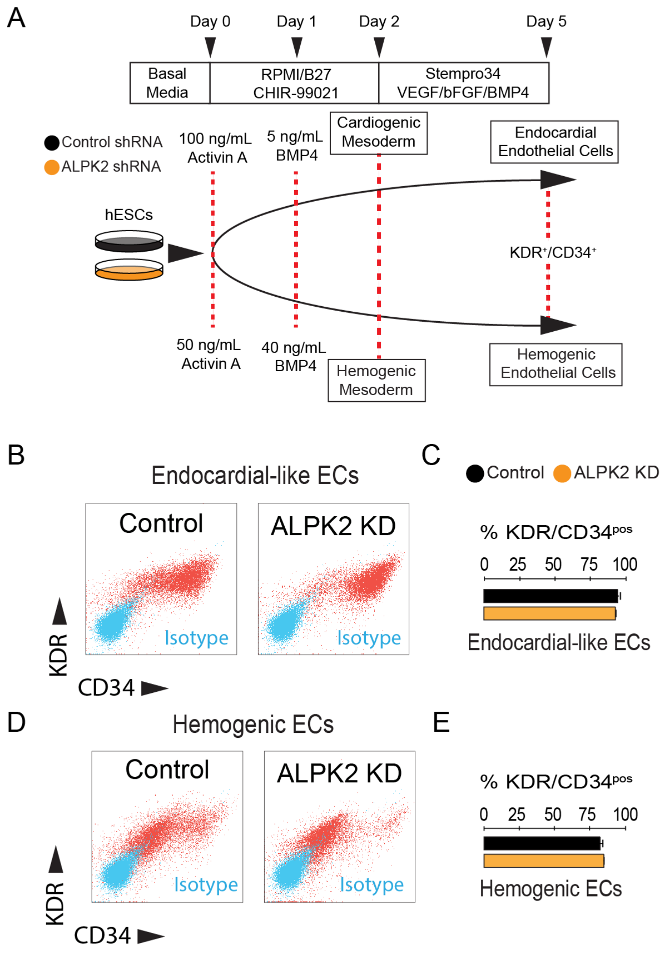

Figure S2: ALPK2 is dispensable for human endocardial and hemogenic endothelial cell differentiation. (A) Schematic of the directed differentiation protocols from human embryonic stem cells towards endocardial-like endothelial cells and hemogenic endothelial cells following control or ALPK2 shRNA transfection. (B, D) Representative flow cytometry scatter plots (B, D; y-axis; VEGF receptor 2 (KDR/FLK1), x-axis; CD34) and quantification (C, E) of control and ALPK2 shRNA knockdown endocardial-like and hemogenic endothelial progenitor cell populations at day 5 of the protocol illustrated in panel A. N=3 biological replicates, Data are mean ±SEM, data were not determined significantly significant by Students t-test. Related to figure 4

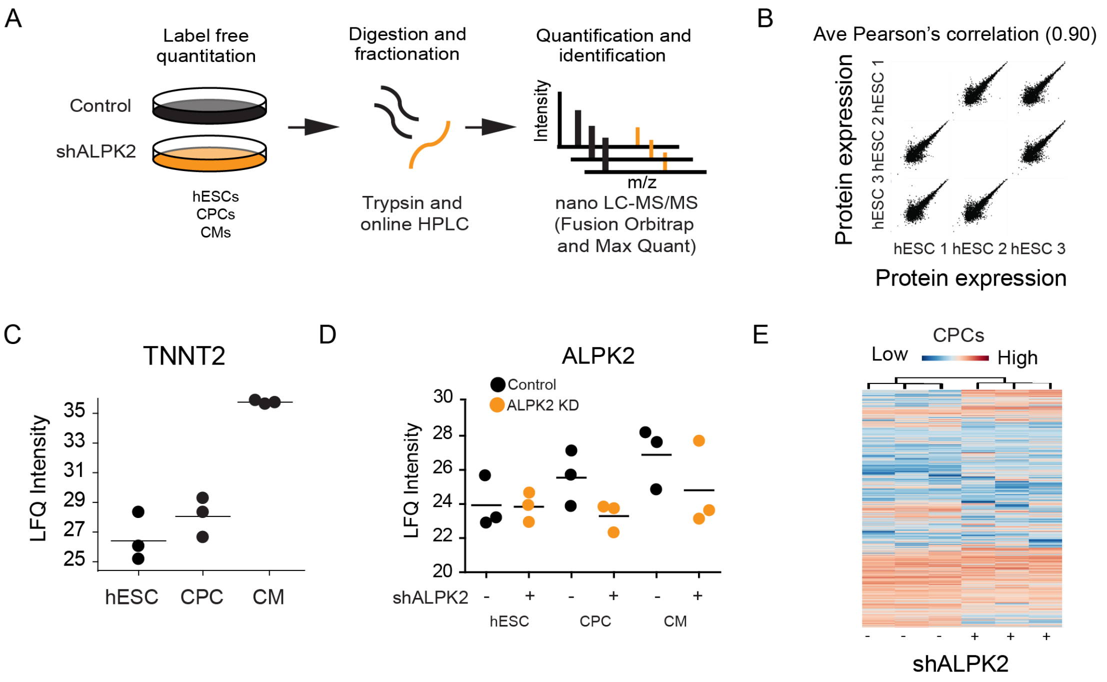

Figure S3: Label free quantitative (LFQ) proteomics of control and ALPK2 shRNA human embryonic stem cells (hESC) differentiated towards cardiac progenitor cells (CPC) and cardiomyocytes (CM). (A) Schematic of LFQ proteomic workflow and reproducibility (B). (C) Protein expression (LFQ intensity) of TNNT2 in hESCs, CPCs and cardiomyocytes (CM). (D) ALPK2 expression over time course cardiomyocyte differentiation and following ALPK2 knockdown. (E) Hierarchical clustering of total protein expression by heat map analysis. Data is representative of 2 biological replicates that were pooled, processed and measured in triplicate. Data were not determined significantly significant by Students t-test. Related to figure 4.

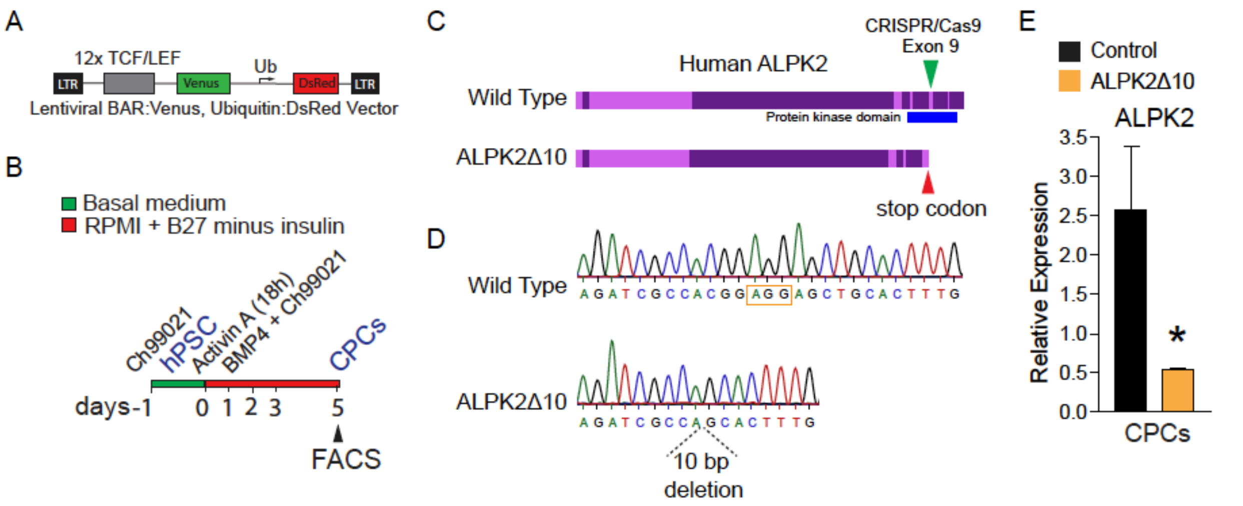

Figure S4: β-catenin-activated-reporter (BAR) assay and CRISPR/Cas9 mutagenesis in human embryonic stem cells (hESC). (A) Depiction of BAR construct to express venus during active β-catenin binding to TCF/LEF elements. (B) Differentiation protocol used to survey BAR activity. (C) Schematic of human ALPK2 locus and CRISPR/Cas9 mutagenesis and (D) Sanger sequencing of wild type (upper) and mutant ALPK2 (ALPK2Δ10, lower) and RT-qPCR (E) for *ALPK2* in wild type and ALPK2Δ10 cardiac progenitor cells derived from human pluripotent stem cells. N=3 biological replicates and data are displayed as mean±SEM. * = p≤0.05. Related to figure 5.

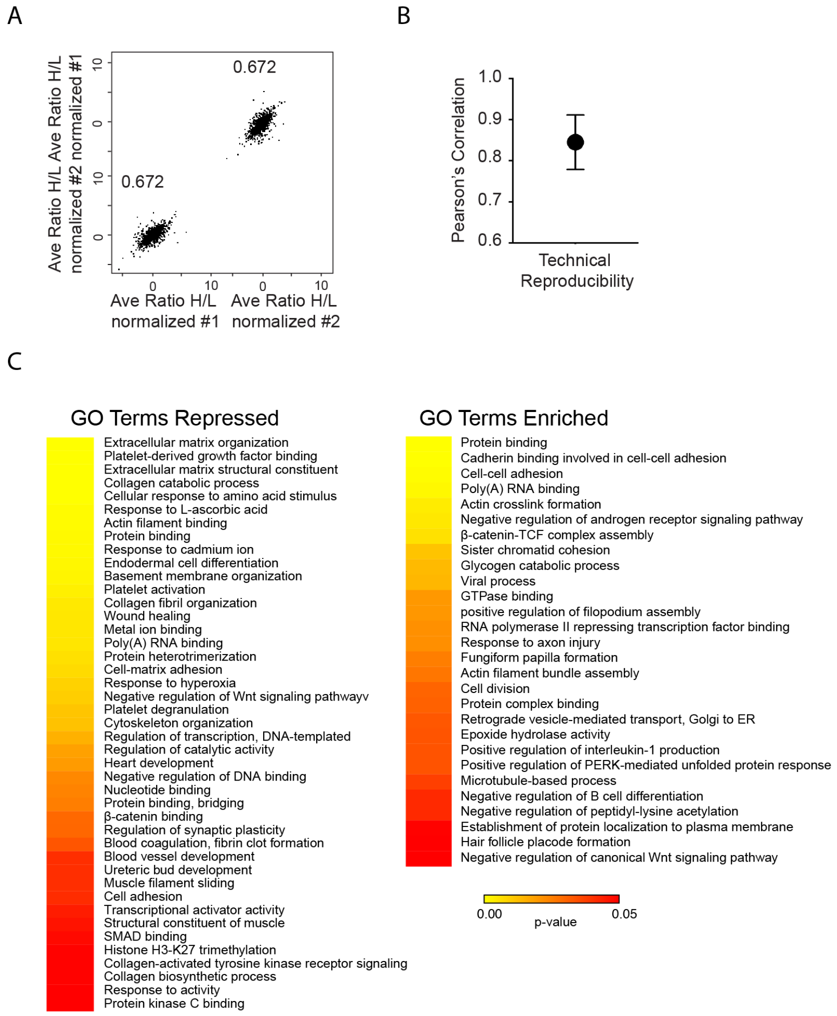

Figure S5: SILAC proteomics reproducibility and GO term analysis. (A, B) Pearson's correlation between biological replicates. (C) Gene ontology (GO) enrichment analysis of upregulated and downregulated proteins as a result of ALPK2 mutagenesis in cardiac progenitor cells derived from human embryonic stem cells. Related to figure 6.

SUPPLEMENTAL TABLES

Table S1: Human and zebrafish oligonucleotides using for gene targeting and RT-qPCR. Related to Figures 1, 2, 3, 4, and 5.

Table S2: All proteins identified by label-free quantitative (LFQ) proteomics. Related to Figure 4

Table S3: ALPK2 regulated proteins identified by label-free quantitative (LFQ) proteomics in cardiac progenitor cells. Related to Figure 4

Table S4: GO Terms enriched by ALPK2 knockdown identified by label-free quantitative (LFQ) proteomics in cardiac progenitor cells. Related to Figure 4.

Table S5: GO Terms repressed by ALPK2 knockdown identified by label-free quantitative (LFQ) proteomics in cardiac progenitor cells. Related to Figure 4.

Table S6: All proteins identified by stable isotope labelling of amino acids in cell culture (SILAC) proteomics. Related to Figure 6.

Table S7: GO Terms enriched in ALPK2Δ10 cardiac progenitor cells identified by stable isotope labelling of amino acids in cell culture (SILAC) proteomics. Related to Figure 6.

Table S8: GO Terms repressed in ALPK2Δ10 cardiac progenitor cells identified by stable isotope labelling of amino acids in cell culture (SILAC) proteomics. Related to Figure 6.

Table S9: All phosphopeptides identified and differentially phosphorylated in ALPK2Δ10 cardiac progenitor cells identified by stable isotope labelling of amino acids in cell culture (SILAC) proteomics. Related to Figure 6.

REFERENCES

Burns, C.G., Milan, D.J., Grande, E.J., Rottbauer, W., MacRae, C.A., and Fishman, M.C. (2005). High-throughput assay for small molecules that modulate zebrafish embryonic heart rate. Nat Chem Biol *1*, 263-264.

Hofsteen, P., Plavicki, J., Johnson, S.D., Peterson, R.E., and Heideman, W. (2013). Sox9b is required for epicardium formation and plays a role in TCDD-induced heart malformation in zebrafish. Mol Pharmacol *84*, 353-360.

Hofsteen, P., Robitaille, A.M., Chapman, D.P., Moon, R.T., and Murry, C.E. (2016). Quantitative proteomics identify DAB2 as a cardiac developmental regulator that inhibits WNT/β-catenin signaling. Proc Natl Acad Sci U S A.

Kikuchi, K., Gupta, V., Wang, J., Holdway, J.E., Wills, A.A., Fang, Y., and Poss, K.D. (2011). tcf21+ epicardial cells adopt non-myocardial fates during zebrafish heart development and regeneration. Development *138*, 2895-2902.

Mably, J.D., Mohideen, M.A., Burns, C.G., Chen, J.N., and Fishman, M.C. (2003). heart of glass regulates the concentric growth of the heart in zebrafish. Curr Biol *13*, 2138-2147.

Moreno-Mateos, M.A., Vejnar, C.E., Beaudoin, J.D., Fernandez, J.P., Mis, E.K., Khokha, M.K., and Giraldez, A.J. (2015). CRISPRscan: designing highly efficient sgRNAs for CRISPR-Cas9 targeting in vivo. Nat Methods *12*, 982-988.

Paffett-Lugassy, N.N., and Zon, L.I. (2005). Analysis of hematopoietic development in the zebrafish. Methods Mol Med *105*, 171-198.

Paige, S.L., Thomas, S., Stoick-Cooper, C.L., Wang, H., Maves, L., Sandstrom, R., Pabon, L., Reinecke, H., Pratt, G., Keller, G., *et al.* (2012). A temporal chromatin signature in human embryonic stem cells identifies regulators of cardiac development. Cell *151*, 221-232.

Palpant, N.J., Hofsteen, P., Pabon, L., Reinecke, H., and Murry, C.E. (2015a). Cardiac development in zebrafish and human embryonic stem cells is inhibited by exposure to tobacco cigarettes and e-cigarettes. PLoS One *10*, e0126259.

Palpant, N.J., Pabon, L., Roberts, M., Hadland, B., Jones, D., Jones, C., Moon, R.T., Ruzzo, W.L., Bernstein, I., Zheng, Y., *et al.* (2015b). Inhibition of β-catenin signaling respecifies anterior-like endothelium into beating human cardiomyocytes. Development.

Palpant, N.J., Wang, Y., Hadland, B., Zaunbrecher, R.J., Redd, M., Jones, D., Pabon, L., Jain, R., Epstein, J., Ruzzo, W.L., *et al.* (2017). Chromatin and Transcriptional Analysis of Mesoderm Progenitor Cells Identifies HOPX as a Regulator of Primitive Hematopoiesis. Cell Rep *20*, 1597-1608.

Plavicki, J., Hofsteen, P., Peterson, R.E., and Heideman, W. (2013). Dioxin inhibits zebrafish epicardium and proepicardium development. Toxicol Sci *131*, 558-567.

Ran, F.A., Hsu, P.D., Wright, J., Agarwala, V., Scott, D.A., and Zhang, F. (2013). Genome engineering using the CRISPR-Cas9 system. Nat Protoc *8*, 2281-2308.

Weidinger, G., Thorpe, C.J., Wuennenberg-Stapleton, K., Ngai, J., and Moon, R.T. (2005). The Sp1-related transcription factors sp5 and sp5-like act downstream of Wnt/beta-catenin signaling in mesoderm and neuroectoderm patterning. Curr Biol *15*, 489-500.

Westerfield, M. (2000). The Zebrafish book. A guide for the laboratory use of zebrafish (*Danio rerio*), 4th ed edn (Eugene, OR: Univ. of Oregon Press).

Yang, X., Rodriguez, M., Pabon, L., Fischer, K.A., Reinecke, H., Regnier, M., Sniadecki, N.J., Ruohola-Baker, H., and Murry, C.E. (2014). Tri-iodo-l-thyronine promotes the maturation of human cardiomyocytes-derived from induced pluripotent stem cells. J Mol Cell Cardiol *72*, 296-304.
